# Measuring System for Determining the Quality of LED Light Sources and an Overview of LED Light Bulbs for Household Use

**DOI:** 10.3390/s22218351

**Published:** 2022-10-31

**Authors:** Matic Markovič, Andrej Orgulan, Primož Sukič

**Affiliations:** Faculty of Electrical Engineering and Computer Science, University of Maribor, Koroška c. 46, 2000 Maribor, Slovenia

**Keywords:** angular distribution of light, fluctuations in the luminous flux, LED light quality, measuring system, Red Pitaya

## Abstract

Modern LED light sources have many advantages, as well as some disadvantages. One of the disadvantages is the pulsating luminous flux, which, in some cases, affects people’s health and well-being negatively. The paper describes the design and making process of a measuring system for determining the quality of LED substitutes for conventional light bulbs and gives an overview of LED light bulbs for household use. The measurement system is controlled using the MATLAB software environment, in which data processing and plotting of the results are also performed. We acquired 59 different LED light bulbs from 37 manufacturers, and performed the measurements. The light bulbs are classified based on the percentage of fluctuations in the luminous flux, and the percentage of deviation of the measured luminous flux compared to the value stated on the packaging by the manufacturer.

## 1. Introduction

The number of light-emitting diode (LED) light bulbs on the market is increasing nowadays. They have almost completely replaced incandescent, energy-saving, and halogen lamps. Consumers are choosing modern LED bulbs because of EU Directives and their many advantages compared to energy-saving light bulbs. LED light bulbs consume less electricity, have a longer lifetime, and are made of less environmentally harmful materials compared to energy-saving light bulbs [1]. In parallel to the many advantages of LED bulbs, research also shows their disadvantages [2]. The evolution of humans and animals has occurred by exposure to light from the Sun. Hot bodies, including the Sun, are the source of an almost constant luminous flux. In recent decades, the use of artificial light sources has increased exponentially, and, consequently, so has the amount of time people are exposed to artificial light. Most artificial light sources intended for general lighting emit a fluctuating luminous flux, including LED light bulbs. There are different LED bulbs of different qualities on the market, which, of course, also has an impact on the quality of the emitted luminous flux. There are some lower-quality ones that have a high percentage of fluctuation in the luminous flux, which can have a negative impact on people’s well-being and health [2].

The fluctuating luminous flux emitted from LED bulbs is caused by the fluctuating electric current through the light bulb. The consequences of exposure to a fluctuating luminous flux have been observed, for example, in night-shift workers, who have reported more frequent headaches, fatigue, and concentration difficulties [3,4]. Fluctuating lighting has a negative impact on healthy people, and an especially strong impact on people with neurological disorders such as photosensitive epilepsy, as well as on individuals with autism [5].

As a part of the project, we reviewed the scientific and technical literature about the negative effects of fluctuating light on humans. We designed and assembled a portable measuring system that enables us to perform multiple measurements for determining the quality of LED substitutes for conventional light bulbs. In this research, we only tested light sources with a maximum bulb diameter of 65 mm. On a photometric bench (measuring system), we determined the minimum distance between the sensor and the light source, so that the solid angle of the light source towards the sensor is within the required limits [6]. Therefore, we have defined a distance of 900 mm between the sensors and the light source, which ensures acceptable measurement uncertainty due to a solid angle greater than 0 sr. The distance between the sensor and the light source (900 mm) is also determined by the length of the standard-size tubes and the space between the center of the light bulb and the first aperture of the tunnel, which provides enough space to rotate the rotary sliding table with the light bulb holder. The measuring system is controlled using the MATLAB software environment, which is also used to plot the results:

- measurement and plot for observing fluctuations in the emitted luminous flux,
- measurement and plot for observing the AC current flowing into the switching converter (LED bulb),
- the illuminance measurement and polar diagram of the angular distribution of luminous intensity,
- plotting the frequency spectrum and spectrogram of the luminous flux signal,
- measurement of the luminous flux emitted by the light source.

After completion of the measurement system, we procured the 59 different light bulb measurements listed below. The results were analyzed, and the LED bulbs were classified based on two parameters: The percentage of fluctuations in the luminous flux, and the deviation of the measured luminous flux compared to the value stated on the packaging.

## 2. Measuring System [7]

**Figure 1:**
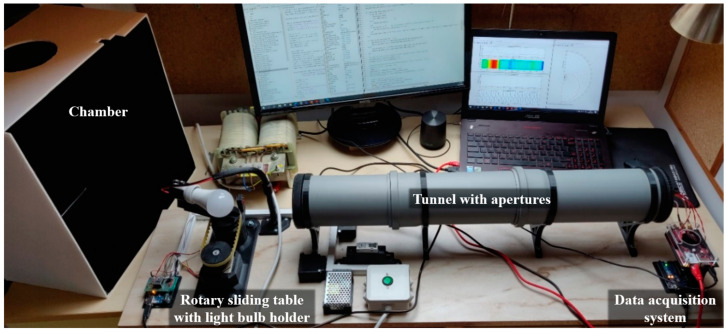
Measuring system.

The measuring system consists mainly of self-developed components (specifically modeled with SolidWorks software and printed with a 3D printer), commercially available components, and a self-developed electronic assembly. Each 3D printed part has its own function and is customized to standard components. Figure 1 shows the measuring system, the chamber designed to prevent the influence of ambient light on the measurements, and the isolating transformer.

### 2.1. Rotary Sliding Table with Light Bulb Holder

**Figure 2:**
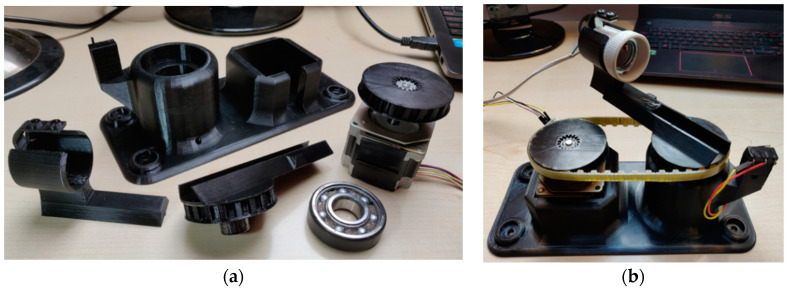
(**a**) Components of the rotary sliding table; (**b**) Assembled rotary sliding table.

The rotary sliding table of the measuring system consists of a 3D printed base that serves to mount the stepper motor, the radial roller bearing, and the limit switch in a specific position (Figure 2).

**Figure 3:**
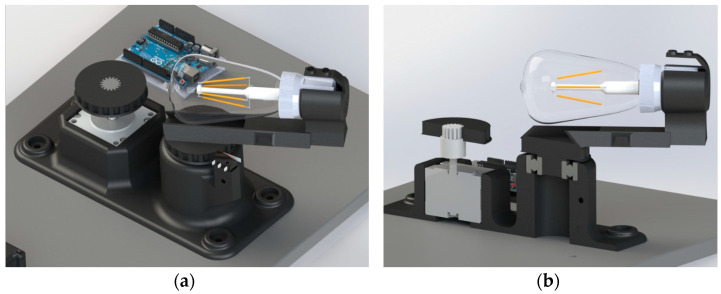
(**a**) 3D model of the light bulb rotation system; (**b**) Section view of the light bulb rotation system.

During the measurements, the rotary sliding table with the light bulb holder is rotated precisely by a stepper motor. We used the NEMA 23 stepper motor, which rotates the 3D-printed sliding table, the light bulb holder, the light bulb socket, and the light bulb. The stepper motor has a 16-tooth gear attached to the end of the shaft. A driving pulley with internal toothing is attached to the gear. It transmits the angle of rotation to the driven pulley using a toothed belt. The pulleys are identical, with equal diameters, modules, and the number of teeth. The mounting of the listed components in the 3D printed base ensures a constant center-to-center distance between the pulleys (100.5 mm) and a low pre-tension force required for the use of a toothed belt. The transmission of the rotation using the toothed belt ensures smooth running without belt slippage. During measurements, the stepper motor rotates the sliding table with the light bulb from 0° to 180° with a step of 3.6°, which means that the stepper motor performs a rotation of two steps during the measurements (step angle of the stepper motor: 1.8°). A series of 51 measurements from a rotation angle of 0° to 180° was found to be sufficiently frequent for plotting a polar diagram of the angular distribution of the luminous intensity. An adjustable sliding table is modeled on the upper side of the driven pulley, into which the holder for the E27 light bulb socket is inserted (Figure 3). The socket can be moved along the sliding table, allowing measurements of light bulbs of various dimensions.

### 2.2. Tunnel with Apertures

The tunnel with the apertures serves as a filter for the light heading toward the sensor. It only lets through direct light from the light source entering perpendicularly to the illuminance sensor located at the end of the tunnel. The tunnel consists of PVC pipes painted matt black on the inner walls to maximize light absorption. The larger surfaces of the inner walls of the pipes and the apertures are also covered with a fine black velour flocking material which absorbs the light very well. The tunnel is mounted on printed supporting legs. Thus, the longitudinal centerline of the tubes is at the same height as the center of the light bulb, the illuminance sensor, and the photodiode at the end of the tunnel.

**Figure 4:**
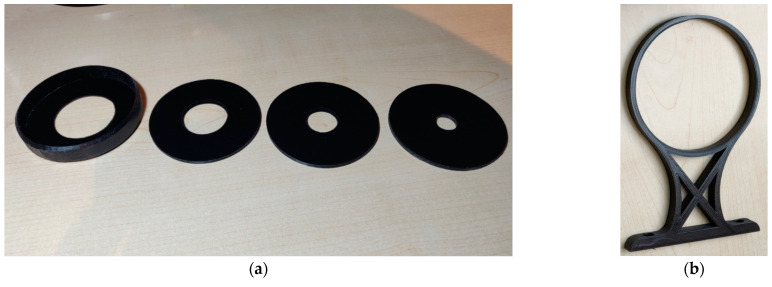
(**a**) Tunnel apertures with progressive inner openings diameters; (**b**) Tunnel supporting leg.

**Figure 5:**
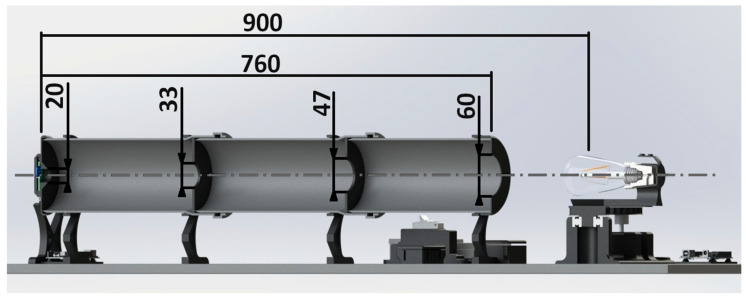
Cross-section view of the measuring system with dimensions [mm].

The purpose of the apertures is to prevent the progression of reflected light. The transmittance of only direct light is achieved by using several apertures in series. The reflected light encounters the barriers and the absorbing material, where it is largely absorbed, and thus reaches a negligible amplitude after a few reflections. It is important that only direct light falls on the sensor. The openings in the apertures are progressive, as shown in (Figure 4 and Figure 5). On the side of the tunnel where the light enters, the aperture diameter is the largest. The diameters decrease towards the sensors at the end of the tunnel. The dimensions of each aperture, the length of the tunnel, and the distance from the center of the light bulb to the sensors at the end of the tunnel are shown in (Figure 5). The dimensions are in millimeters.

### 2.3. Light Bulb Power Cable Stand

**Figure 6:**
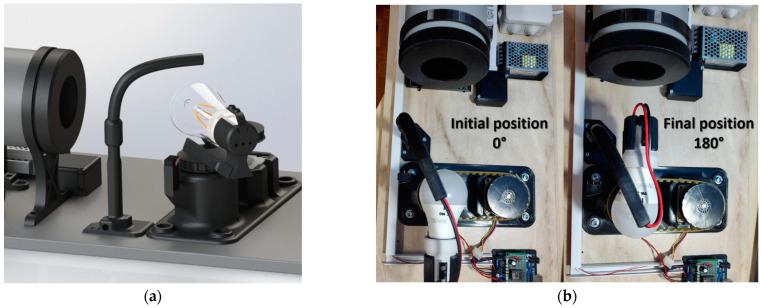
(**a**) Light bulb power cable stand; (**b**) The initial and final positions of the light bulb for plotting the polar diagram of the angular distribution of luminous intensity.

The light bulb power cable stand has been designed so that the cable is routed above the apex of the radiating part of the light bulb and the driven pulley. This ensures that the distance of the cable between the socket and the cable stand does not change, and, consequently, the cable has no effect on the rotation of the rotary sliding table (Figure 6). The cable is also raised above the light bulb, to ensure that it does not pass between the light bulb and the tunnel during the measurement, which would distort the measurement. The stand is positioned next to the limit switch, allowing the rotation of the sliding table to the limit switch, and the complete measurement to be carried out without interruption.

### 2.4. Chamber for Preventing the Influence of Ambient Light on Measurements

**Figure 7:**
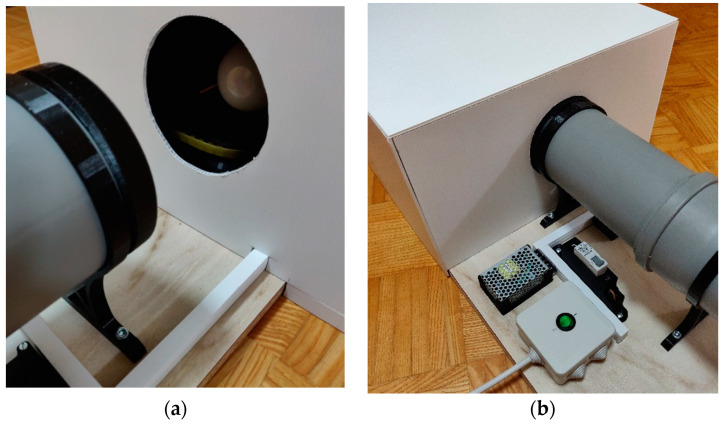
Installing the chamber on the measuring system: (**a**) Before installation; (**b**) After installation.

The chamber (Figure 7) was designed to prevent the influence of ambient light on the measurements, and covers the light bulb rotation system, the power cable stand, and the first aperture of the tunnel completely. This ensures that the light bulbs and sensors are exposed to the same conditions during measurements.

**Figure 8:**
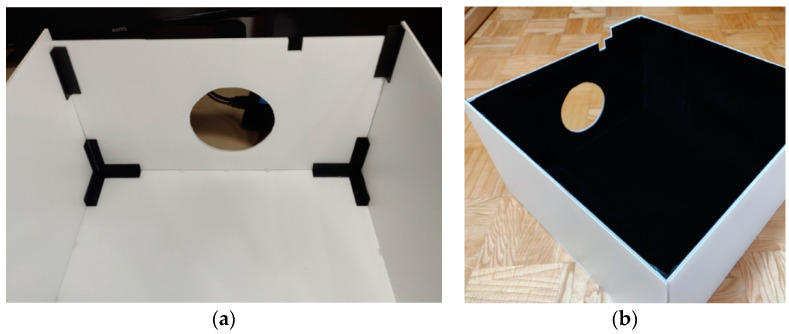
(**a**) Assembling the chamber; (**b**) Inner walls of the chamber covered with black velour flocking material.

The chamber is made of 5 mm thick white corrugated polypropylene panels that reflect external light. The panels are connected to one another by 3D-printed profiles (Figure 8). The inner walls of the chamber are covered with black velour flocking material, which absorbs light very well.

### 2.5. Microcontroller and Multifunctional Measurement Device Red Pitaya Holder

**Figure 9:**
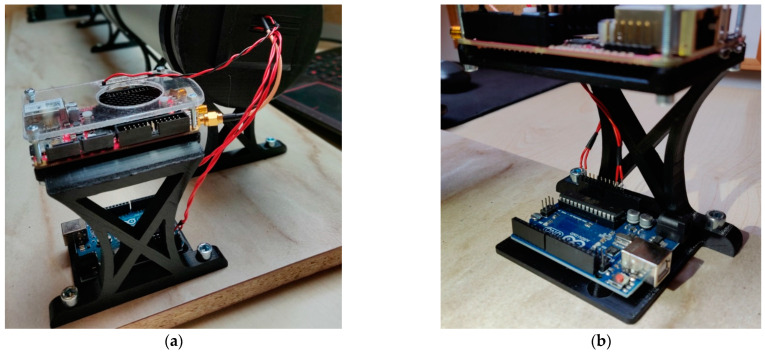
Microcontroller and multifunctional measurement device Red Pitaya holder: (**a**) Multifunctional measurement device Red Pitaya—above; (**b**) Microcontroller Arduino—below.

The Red Pitaya multifunctional measurement device and the microcontroller Arduino are located at the end of the tunnel, close to the sensors. They are mounted on a custom-designed, 3D-printed holder (Figure 9). The holder is designed so that the Red Pitaya is raised from the base by 90 mm. This ensures that the distance is minimized between the electronic circuit for observing fluctuations in the luminous flux with the photodiode and the fast analog input of the Red Pitaya (IN1).

### 2.6. Electronic Assembly for Stepper Motor Control

**Figure 10:**
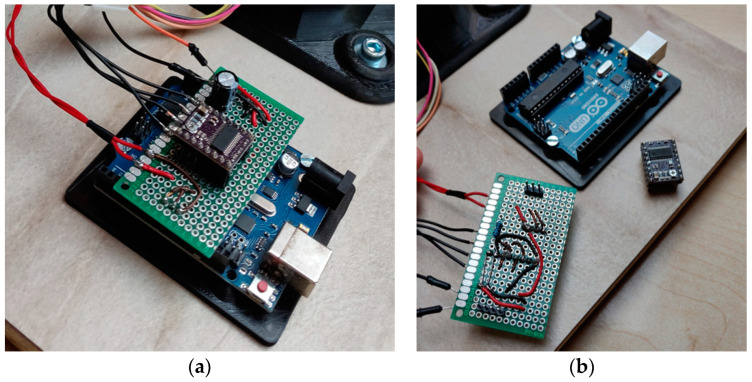
(**a**) The electronic assembly developed to control the stepper motor; (**b**) Arduino microcontroller, stepper motor driver DRV8825, and the electronic assembly.

To measure the luminous flux of an LED light bulb at various angles, we have to rotate it around its center. This is performed with a stepper motor, which requires an electronic assembly for the controlled and desired rotation of the motor. At the start of the measurements, the sliding table with the light bulb is rotated clockwise to the limit switch. Then the stepper motor rotates the sliding table counterclockwise by 90° to position the light bulb in the initial position to perform the measurement. The rotary sliding table rotates the light bulbs in steps, and the measuring system performs the measurements. During each measurement, a stepper motor rotates the sliding table by 3.6° in a counterclockwise direction. From an initial position at 0° to the final measurement at 180°, the measuring system performs 51 measurements. The electronic assembly developed to control the stepper motor with the DRV8825 stepper motor driver is shown in (Figure 10) [8].

The stepper motor is controlled by the MATLAB program, which sends a command to rotate the light bulb holder through a serial connection to the Arduino microcontroller. MATLAB sends an integer “char” value via serial communication. Arduino receives the value on the serial input and stores it in a dedicated variable. In the program which runs on the Arduino, four conditions are written which check the value in the variable, and so the part of the program that was requested is executed (enable the stepper motor, disable stepper motor, rotate the light bulb to the initial position, rotate the sliding table by 3.6°).

### 2.7. Electronic Assembly for Illuminance Measurement

The goal is to measure the angular distribution of the luminous intensity and plot the measurements in a polar diagram. This is achieved with an illuminance sensor, which measures the values at different angles of the light bulb. The measured values are subsequently calculated into luminous intensity.

**Figure 11:**
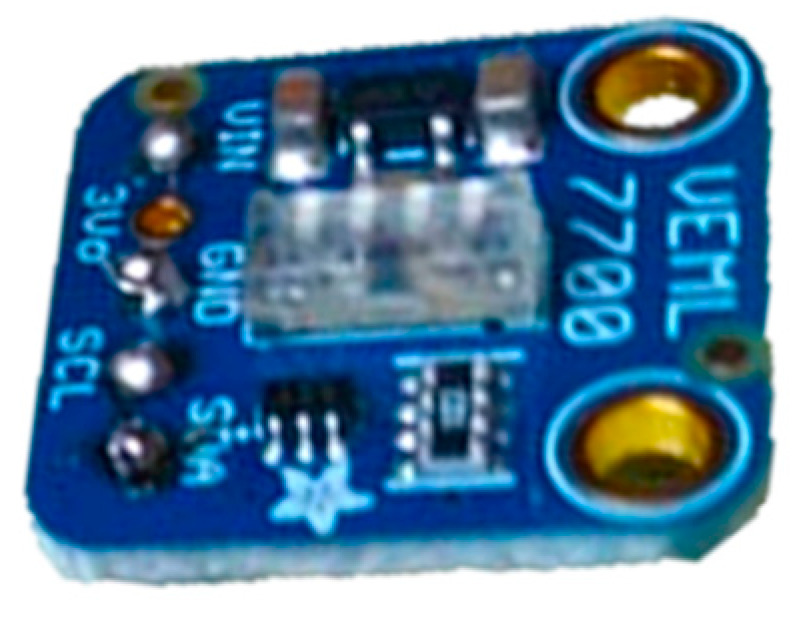
Illuminance sensor VEML7700.

The measuring system is equipped with a luminance sensor from Adafruit, with the designation VEML7700 (Figure 11). It is connected to the I2C bus of an Arduino microcontroller. The illuminance sensor is capable of measuring values between 0 lux and 120,000 lux, with a resolution of 0.0036 lux. In the Arduino integrated development environment, we specified the desired sampling rate of the sensor. The averaging time selected was 800 ms (averaging 80 half-periods of the AC mains frequency) and a gain value set to 1, which determines the expected range of the measured illuminance. The illuminance sensor is connected to the I2C bus of the Arduino microcontroller, which receives an already calculated value in lux. It is fitted in a purpose-made opening of the PCB. The sensor is directed perpendicularly to the light bulb, and receives the light through the last aperture of the tunnel with an inner hole diameter of 20 mm. The Arduino microcontroller sends the received value of illuminance via serial communication to MATLAB. The sensor performs measurements in an interval of 0.8 s.

### 2.8. System for High-Frequency Data Acquisition

The high-frequency data acquisition system consists of the multifunctional measurement device Red Pitaya and a custom-designed electronic assembly. The Red Pitaya’s high-speed analog inputs were used to acquire data for time-course observations of the AC current and luminous flux. The Red Pitaya is connected to MATLAB via an Ethernet connection. The voltage samples at the output of the electronic assembly and the voltage drop across the shunt resistor are acquired using a streaming application on the Red Pitaya measurement device. The following data acquisition parameters were set in the application [9]:

- sampling frequency: 300 kHz,
- save measured samples in audio file format (WAV),
- save samples from both analog inputs,
- resolution of input channels: 16 bits,
- data transmission protocol: TCP,
- number of acquired samples: 150,000 (duration of the acquisition: 0.5 s),
- IP address of the measurement device Red Pitaya.

Example of a command line for data acquisition:

System (‘rpsa_client.exe -h 169.254.33.31 -p TCP -f ./ -t wav -s 150,000’);

We used the streaming application “rpsa_client.exe”, specified the IP address of the device, selected the TCP data transfer protocol, chose the WAV data storage format, and specified the desired number of samples to be captured: 150,000.

### 2.9. Electronic Circuit for Observing Fluctuations in the Luminous Flux

**Figure 12:**
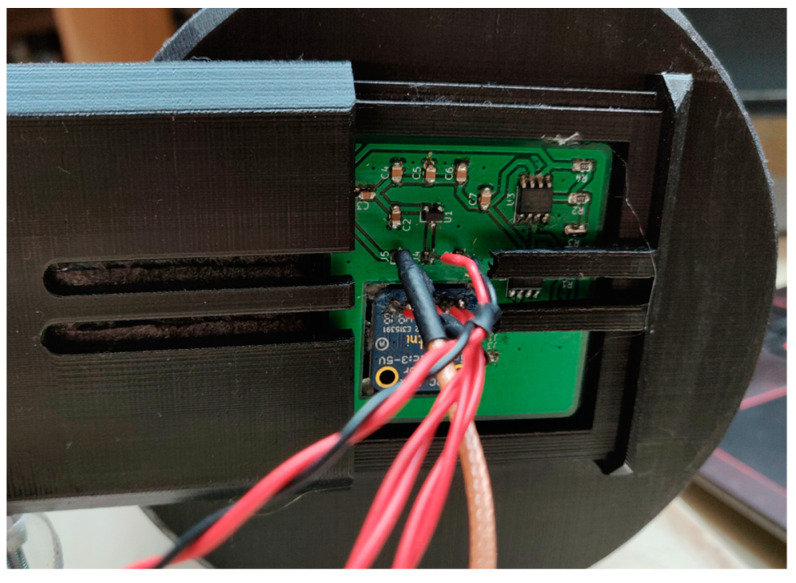
Designed PCB for observing fluctuations in the luminous flux.

LED light sources do not emit a constant luminous flux, which is caused by an alternating electric current through the light source. The light bulbs emit a fluctuating luminous flux (flickering) which, in some circumstances, can affect the health and well-being of persons exposed to such lighting adversely. LED light bulbs from different manufacturers differ in the manufacturing quality and in the percentage of fluctuation in the luminous flux. The higher the percentage of fluctuation, the greater the impact on people exposed to the light. The printed circuit board designed for observing fluctuations in the luminous flux is shown in (Figure 12).

**Figure 13:**
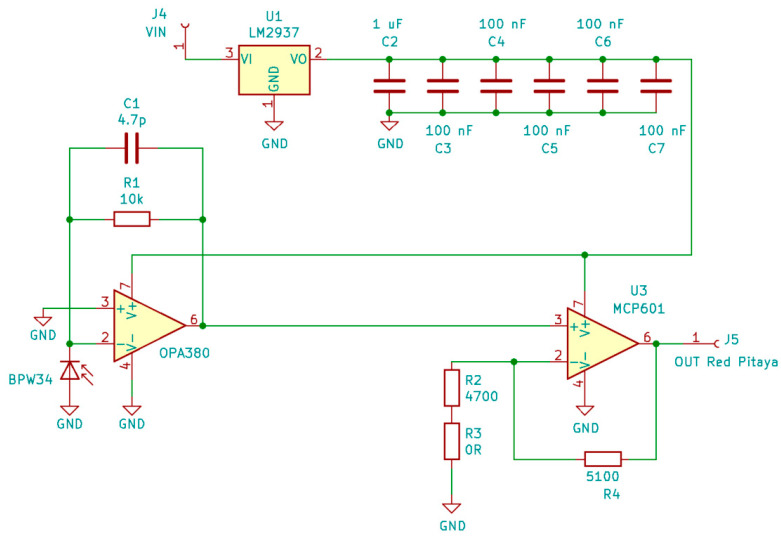
The electronic circuit diagram of the sensor for observing fluctuations in the luminous flux.

The electronic circuit for observing fluctuations in the luminous flux was simulated in the LTspice simulation software and designed in KiCAD. Figure 13 shows the electric circuit schematic. The circuit includes a fast photodiode BPW34, which absorbs photons and generates an electric current. The generated electric current is proportional to the power of the incident light. The transimpedance amplifier OPA 380 transforms the electric current into a measurable electric voltage. The voltage values are quite small, and are therefore amplified in the 1 V range with the operational amplifier MCP601, which is the measurable voltage range of the Red Pitaya analog input. The amplifiers are powered by a linear voltage regulator [10].

**Figure 14:**
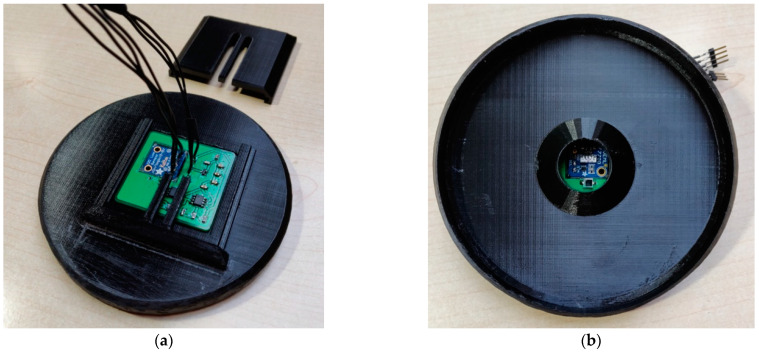
Tunnel lid with positioned sensors: (**a**) The outer side where the PCB with the sensors is placed; (**b**) The inner side, which shows the 20 mm diameter aperture.

The designed PCB is placed in a specially modeled and 3D-printed tunnel lid (Figure 14), which positions the sensors at the same height as the center of the light bulb and the apertures. The lid also prevents the influence of ambient light on the measurements. The maximum amplitude-frequency of fluctuations of the luminous flux is twice the value of the main frequency. The percentage of fluctuation with respect to the measured peak value is also calculated from the measurement.

### 2.10. Electric Circuit for Measuring the Electric Current through a Light Bulb

**Figure 15:**
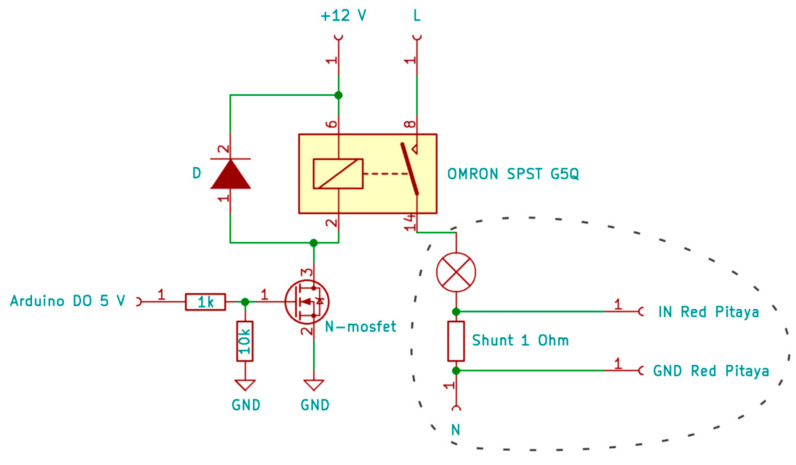
Electric circuit diagram for measuring the electric current through a light bulb (circled area).

The measured current is the AC current of the mains voltage. The measurement of the electric current through the light bulb is performed using a shunt resistor connected in series with the light bulb as seen in the Figure 15. The voltage drop across the shunt resistor is measured with respect to time. The data acquisition is performed using a Red Pitaya measurement device with a sampling frequency of 300 kHz.

**Figure 16:**
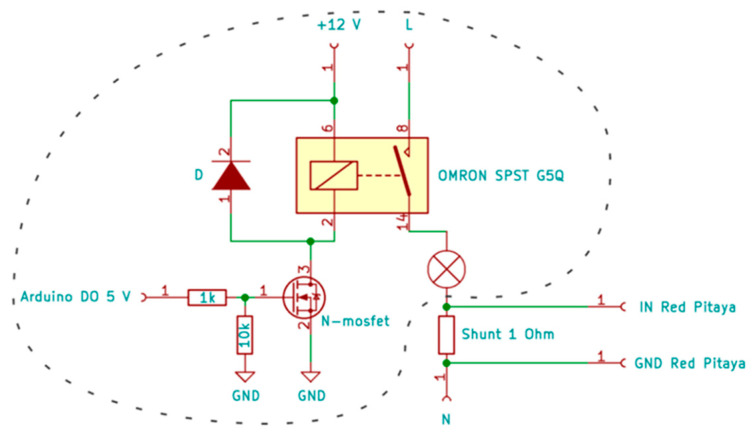
Electric circuit diagram for switching the light bulb ON/OFF (circled area).

The measuring system was used to perform measurements on 59 LED light bulbs. Due to the large number of selected light bulbs, we wanted to automate the measurements as much as possible. It is necessary to switch the light bulb ON before starting the measurements and OFF after the measurements have been performed, which is much easier if this can be performed by software. This also ensures that the light bulbs are measured under the same conditions. MATLAB sends an integer “char” value via serial communication to the Arduino microcontroller. A program that runs on an Arduino microcontroller includes two conditions, that check the received value and executes the required part of the program (switch power ON, switch power OFF). Figure 16 shows a schematic of the designed electrical circuit. The circuit switches the light bulb ON and OFF using a relay.

## 3. Results and Discussion

The purpose of the LED light bulbs’ measurements was to determine which LED light bulbs are good quality according to two parameters: Low fluctuation of the luminous flux compared to the maximum measured amplitude, and if the measured value of the luminous flux complies with the characteristics stated on the packaging. The measured lamps were classified according to these parameters.

**Figure 17:**
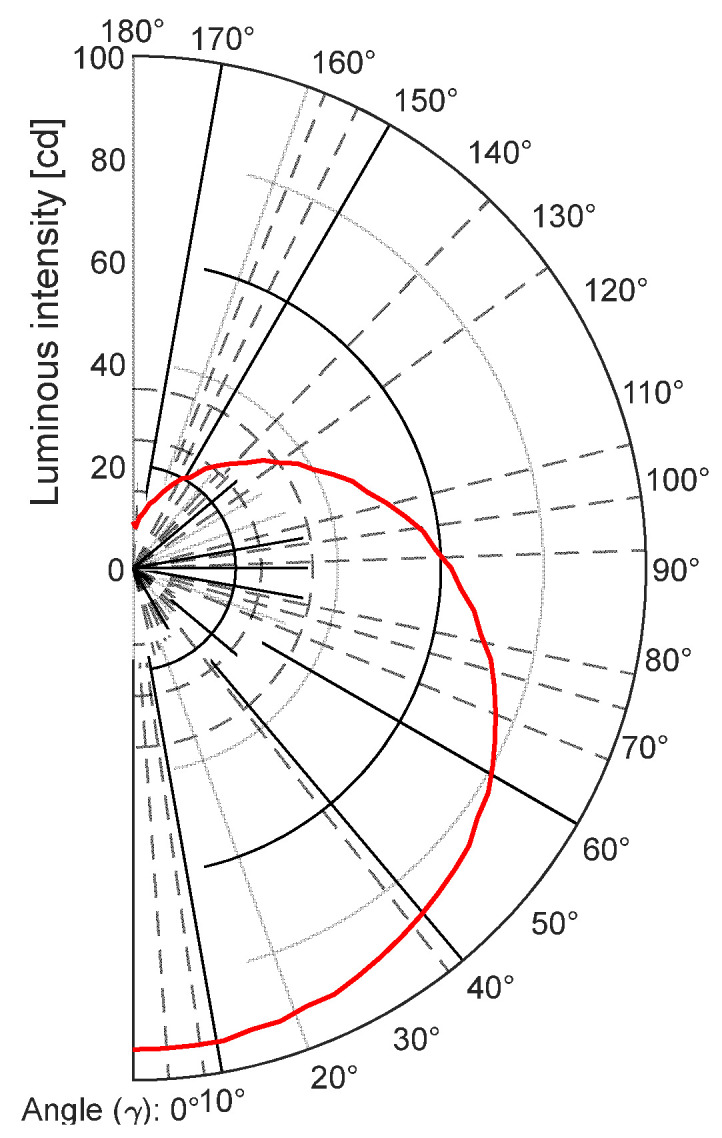
Polar diagram of the angular distribution of the luminous intensity.

Below is an example of the measurement results of an LED light bulb. Figure 17 shows a polar diagram of the angular distribution of the luminous intensity [11,12].

**Figure 18:**
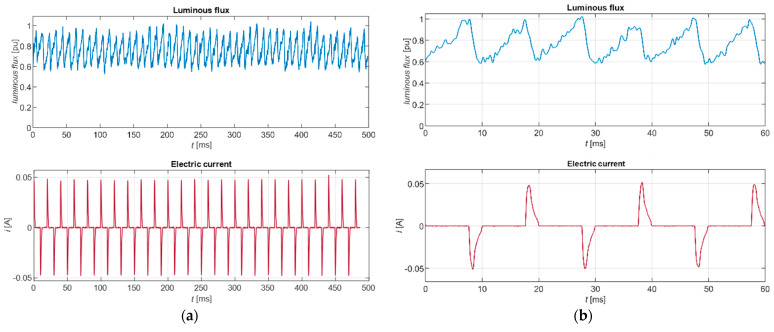
Time course of the luminous flux and electric current: (**a**) Time course up to 500 ms; (**b**) Time course up to 60 ms.

Figure 18 shows the time course of the emitted luminous flux and the electric current flowing through the light bulb. The absciss of the plot is the time axis, from the beginning of the measurement until 500 ms. The absciss axes of the two plots are coordinated. The fluctuation of the luminous flux is calculated and plotted in a per-unit system, giving a clear visual indication of the proportion of fluctuation relative to the maximum measured luminous flux.

**Figure 19:**
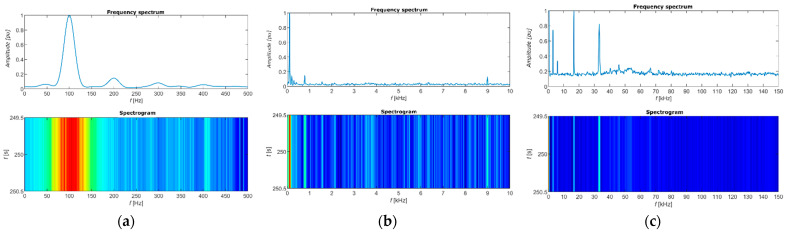
Signal analysis in the frequency domain: (**a**) Frequency range up to 500 Hz; (**b**) Frequency range up to 10 kHz; (**c**) Frequency range up to 150 kHz.

Figure 19 shows the analysis of the luminous flux signal in the frequency domain. We used the MATLAB application Signal Analyzer and a function to perform the Fast Fourier Transform algorithm to plot it. In plot (a) of (Figure 19), we see that the maximum spectral power is at a frequency of 100 Hz, which means in each half period of the mains voltage, current flows into the light bulb. We plotted the FFT in the frequency range up to 500 Hz to show the amplitudes of frequencies that are in the human perceptible frequency range, which means that there is a possibility that the fluctuation of the light current may affect human health and well-being. In plot (c), the range up to 150 kHz, we see the switching frequency of the LED light bulb’s converter. In the spectrograms, frequencies with maximum spectral power are coloured red. The opposite applies for frequencies coloured blue.

### 3.1. List of Measured LED Light Bulbs

The measurements were performed on 59 different LED light bulbs from 37 different manufacturers. All the light bulbs are listed in Table 1, with the light bulb’s code number, manufacturer’s name, and basic technical information: wattage, luminous flux, and the color temperature of the emitted light.

### 3.2. Classification of LED Light Bulbs Based on the Percentage of Fluctuations in the Luminous Flux

**Figure 20:**
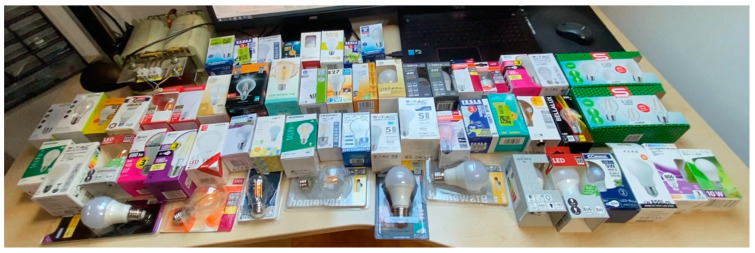
59 measured LED light bulbs.

In (Table 2), the LED light bulbs are ranked in descending order of the percentage of fluctuation with respect to the peak measured luminous flux, from the bulb with the lowest percentage value to the light bulb with the highest percentage value. In this way, they are ranked from the best to the worst measured light bulb. Figure 20 shows the 59 measured LED light bulbs.

The value of the percentage was calculated without considering the 1% of the maximum and minimum measured values, which eliminated a large part of the noise. The results are relative values and not absolute values. The developed measuring system is not certified and calibrated by an accredited institution. Therefore, we have opted for a comparative method with relative proportions to minimize the error of the measuring system. The measurement results of the fluctuation percentage of the luminous flux relative to the peak measured value are presented in a way that the light bulb with the lowest percentage of fluctuation has a value of 0%, and represents the reference light bulb. For the other light bulbs, the percentage value is the relative value with respect to the light bulb with the lowest percentage value. We have chosen to list the top third of LED light bulbs according to the chosen parameter. For light bulbs that do not belong to the top third, we have only stated the calculated percentage of fluctuations. In this way, we achieved the main objective of the project, which was to provide consumers with a list of the LED light bulbs that are ranked in the top third of the tested light bulbs according to the selected parameter. Consumers would not benefit from the listing of light bulbs that did not rank in the top third.

### 3.3. Classification of LED Light Bulbs Based on the Percentage of Deviation of the Measured Luminous Flux Value from the Luminous Flux Value Stated on the Packaging

In (Table 3) LED light bulbs are ranked in descending order according to the percentage of deviation of the measured luminous flux from the luminous flux stated on the packaging. The developed measuring system is not certified by an accredited institution. The measurement of the illuminance has been calibrated with a calibrated reference LED light bulb. The reference measurement in the laboratory and with our measuring system was performed on the same light bulb. Due to the asymmetry of the light bulb, the measurements were performed in the same plane as the lamp. Some light bulbs have a positive percentage of deviation, which means that they emit more luminous flux than stated on the packaging. We have chosen to list the top third of LED light bulbs according to the chosen parameter. For light bulbs that do not belong to the top third, we have only stated the calculated percentage of deviation. In this way, we achieved the main objective of the project, which was to provide consumers with a list of the LED light bulbs that are ranked in the top third of the tested light bulbs according to the selected parameter. Consumers would not benefit from the listing of light bulbs that did not rank in the top third. The measurement was not performed axially, they were performed in the C plane. For the purpose of simplification, we have assumed that measured LED light bulbs are axially symmetrical, which is not correct for all types of light bulbs. Some light bulbs may have measurement deviations.

## 4. Conclusions

During the work on the project, we designed and assembled a measuring system for evaluating the quality of the luminous flux of LED light sources for households available on the market. We performed measurements of 59 LED light bulbs of different types. The results of the measurements were analyzed in the MATLAB software environment and classified according to two key parameters: The percentage of fluctuation compared to the maximum measured luminous flux value, and the deviation of the luminous flux value emitted by the light bulb compared to the value stated by the manufacturer.

The conclusions of the measurements are, that the luminous flux of the LED light bulbs for households and that the luminous flux of the LED light bulbs fluctuates with twice the mains frequency and with the switching frequency of the light bulb’s converter. It was concluded that there is a deviation of the value of measured luminous flux emitted by the light bulb from the value stated by the manufacturer on the packaging. The results show large variations between different light bulbs. A significant number of light bulbs have a negative deviation, a significant fluctuation of luminous flux, and do not match the luminous flux value stated on the packaging. On the other hand, there is a significant number of good quality light bulbs that have minimal deviation and percentage of fluctuations of luminous flux. In this way, we can choose better LED light bulbs.

A video showing the operation of the measuring system is available on YouTube: https://www.youtube.com/watch?v=9aiAIsVnVpQ&ab_channel=MaticMarkovi%C4%8D, accessed on 7 September 2022.

The program codes used are available on GitHub: https://github.com/MaticMarkovic/Measuring-system-for-determining-the-quality-of-LED-light-sources, accessed on 7 September 2022.

3D models (Solidworks files) are available on GrabCAD: https://grabcad.com/library/measuring-system-for-determining-the-quality-of-led-light-sources-3d-model-1, accessed on 7 September 2022.

3D models (STL files) are available on Thingiverse: https://www.thingiverse.com/thing:5038490, accessed on 7 September 2022.

## Figures and Tables

**Table 1:** List of measured LED light bulbs.

Code nr.	Manufacturer	Wattage [W]	Luminous Flux [lm]	Color Temperature [K]
1	SYLVANIA (ToLEDo Retro GLS)	7	806	2700
2	SYLVANIA (ToLEDo GLS)	8.5	806	2700
3	LIGHTWAY	11	806	2700
4	XAVAX	9	806	2700
5	BELLALUX	7	806	2700
6	EMOS (LED filament)	8	1060	2700
7	EMOS (LED A60, step dimmable)	10	880	2700
8	EMOS (LED Classic)	10	806	2700
9	OSRAM (LED value classic A60)	8.5	806	2700
10	OSRAM (LED star classic A60, retrofit)	7	806	2700
11	OPTONICA	10	820	4500
12	WELL LIGHT (Basic)	10	750	3000
13	PHILIPS (CorePro LEDbulb)	9	806	2700
14	AVIDE	8	640	3000
15	OSRAM (LED superstar P25 advanced)	4	250	2700
16	OSRAM (Parathom classic A100)	11	1521	2700
17	BOXXX	5.5	470	3000
18	HOMEWARE (LED filament)	6	550	2700
19	HOMEWARE	6	470	3000
20	V-TAC (LED A60 bulb)	9	806	4000
21	V-TAC (Pro Samsung LED chip)	9	806	4000
22	V-TAC (Dimmable filament A67 bulb)	8	700	2700
23	V-TAC (LED R80 bulb)	10	800	4000
24	ETA	7	640	2700
25	ETA (LED filament)	8	1055	2700
26	TESLA (LED filament)	4	470	2700
27	TESLA (reflektorska)	7	560	3000
28	TESLA	7	640	3000
29	TUNGSRAM (LED filament)	4.5	470	2700
30	TUNGSRAM	9	810	2700
31	BLAUPUNKT (G45 LED)	4	470	2700
32	BLAUPUNKT (A60 LED)	8.5	806	3000
33	GOOBAY	9	810	2700
34	GOOBAY (LED filament)	4	450	2700
35	FEROTEHNA	5	400	3000
36	VOLTOLUX (LED filament)	4	470	2700
37	VOLTOLUX	8.5	806	2700
38	SIMPEX (LED filament, dimmable)	8.3	806	2700
39	SIMPEX	9.4	806	2700
40	S-BUDGET	7	806	2700
41	S-BUDGET (LED filament)	7	806	2700
42	COMMEL	9	806	3000
43	TORE	9	806	2700
44	KOBI-LIGHT	10	800	3000
45	KOBI-LIGHT (LED filament, retro)	7	800	2700
46	VP-EL	12	1200	2700
47	GLOBO	6	650	2700
48	GLOBO (LED filament)	7	806	2700
49	HOROZ	8	850	4200
50	ISKRA	5	470	3000
51	ISKRA (LED filament)	4	400	2700
52	EGLO (LED filament, dimmable)	6	806	2700
53	GE LIGHTING (LED filament)	5	590	2700
54	GE LIGHTING	7	470	2700
55	BRILAGI (ECO line A60)	10	900	3000
56	FARO (milky LED)	7	800	2700
57	EMITHOR	5	400	3000
58	EGLO (dimmable)	10	806	3000
59	PAULMANN (LED filament, dimmable)	4.5	470	2700

**Table 2:** Classification of LED light bulbs based on the percentage of fluctuations in the luminous flux.

Ranking	Code nr.	Manufacturer	Percentage of Fluctuations [%]
1.	8	EMOS (LED Classic)	0
2.	27	TESLA (reflektorska)	4.1
3.	23	V-TAC (LED R80 bulb)	5.0
4.	46	VP-EL	5.5
5.	16	OSRAM (Parathom classic A100)	7.6
6.	37	VOLTOLUX	7.8
7.	13	PHILIPS (CorePro LEDbulb)	7.8
8.	32	BLAUPUNKT (A60 LED)	8.3
9.	2	SYLVANIA (ToLEDo GLS)	8.4
10.	43	TORE	8.6
11.	7	EMOS (LED A60, step dimmable)	8.9
12.	42	COMMEL	10.1
13.	58	EGLO (dimmable)	10.8
14.	44	KOBI-LIGHT	10.8
15.	55	BRILAGI (ECO line A60)	11.2
16.	33	GOOBAY	11.5
17.	4	XAVAX	11.5
18.	24	ETA	12.2
19.	9	OSRAM (LED value classic A60)	12.8
20.	/	/	13.0
21.	/	/	13.1
22.	/	/	13.2
23.	/	/	13.2
24.	/	/	13.3
25.	/	/	13.8
26.	/	/	14.0
27.	/	/	14.1
28.	/	/	14.4
29.	/	/	16.7
30.	/	/	17.7
31.	/	/	17.8
32.	/	/	17.9
33.	/	/	19.3
34.	/	/	20.3
35.	/	/	20.5
36.	/	/	20.8
37.	/	/	21.3
38.	/	/	23.2
39.	/	/	23.5
40.	/	/	23.6
41.	/	/	24.1
42.	/	/	24.5
43.	/	/	24.7
44.	/	/	26.2
45.	/	/	26.7
46.	/	/	28.1
47.	/	/	28.8
48.	/	/	29.3
49.	/	/	29.9
50.	/	/	32.7
51.	/	/	34.6
52.	/	/	35.5
53.	/	/	36.6
54.	/	/	53.6
55.	/	/	63.6
56.	/	/	64.6
57.	/	/	65.4
58.	/	/	74.0
59.	/	/	82.4

**Table 3:** Classification of LED light bulbs based on the percentage of deviation of the measured luminous flux value from the luminous flux value stated on the packaging.

Ranking	Code nr.	Manufacturer	Deviation [%]
1.	16	OSRAM (Parathom classic A100)	−0.3
2.	43	TORE	0.6
3.	54	GE LIGHTING	−0.9
4.	38	SIMPEX (LED filament, dimmable)	−1.0
5.	50	ISKRA	1.6
6.	34	GOOBAY (LED filament)	2.3
7.	5	BELLALUX	−3.1
8.	48	GLOBO (LED filament)	−3.3
9.	21	V-TAC (Pro Samsung LED chip)	−3.3
10.	11	OPTONICA	3.5
11.	2	SYLVANIA (ToLEDo GLS)	−4.5
12.	3	LIGHTWAY	4.8
13.	32	BLAUPUNKT (A60 LED)	−5.1
14.	42	COMMEL	−5.7
15.	47	GLOBO	−5.7
16.	23	V-TAC (LED R80 bulb)	−6.3
17.	37	VOLTOLUX	−6.3
18.	19	HOMEWARE	6.5
19.	56	FARO (milky LED)	−7.2
20.	/	/	7.2
21.	/	/	−7.8
22.	/	/	8.0
23.	/	/	8.7
24.	/	/	9.1
25.	/	/	−9.3
26.	/	/	−9.6
27.	/	/	−10.1
28.	/	/	10.1
29.	/	/	−10.6
30.	/	/	−11.2
31.	/	/	−11.5
32.	/	/	11.5
33.	/	/	−12.5
34.	/	/	−13.4
35.	/	/	−13.9
36.	/	/	−14.0
37.	/	/	14.5
38.	/	/	−15.6
39.	/	/	−16.8
40.	/	/	−17.9
41.	/	/	−18.2
42.	/	/	−18.5
43.	/	/	−19.0
44.	/	/	−19.4
45.	/	/	−19.5
46.	/	/	−20.4
47.	/	/	−20.4
48.	/	/	−20.5
49.	/	/	−21.1
50.	/	/	−21.2
51.	/	/	−22.4
52.	/	/	24.7
53.	/	/	−25.8
54.	/	/	−25.9
55.	/	/	30.4
56.	/	/	−30.9
57.	/	/	−41.5
58.	/	/	−42.2
59.	/	/	−47.4

## Data Availability

All the data is contained within the paper.

## References

[1] Udovychenko K.O. (2019). The Advantages and Disadvantages of Using Led Light Bulbs.

[2] Wang Q., Xu H., Gong R., Cai J. (2015). Investigation of visual fatigue under LED lighting based on reading task. Optik.

[3] Serikov Y.A., Nazarenko L.A., Serikova K.S. (2020). Non-Visual Exposure to Light as a Production Factor of the Influence of Lighting of the Working Area on Labor Productivity and Safety of Workers. Metrol. Instrum..

[4] Berzina K., Zicmane I., Podgornovs A., Zhiravecka A., Kuckovskis J., Berzina N.N. The impact of lighting fluctuations on anthropological aspects. Proceedings of the 2018 IEEE International Conference on Environment and Electrical Engineering and 2018 IEEE Industrial and Commercial Power Systems Europe (EEEIC/I&CPS Europe).

[5] Inger R., Bennie J., Davies T.W., Gaston K.J. (2014). Potential Biological and Ecological Effects of Flickering Artificial Light. PLoS ONE.

[6] Sametoglu F. (2011). Construction of Two-Axis Goniophotometer for Measurement of Spatial Distribution of a Light Source and Calculation of Luminous Flux. Acta Phys. Pol..

[7] Markovič M. (2021). Measuring System for Determining the Quality of LED Substitutes for Conventional Light bulbs. Bachelor’s Thesis.

[8] Pololu, Stepper Motor Driver Carrier, High Current. https://www.pololu.com/product/2133.

[9] Lock-in+PID, the Red Pitaya Board. https://marceluda.github.io/rp_lock-in_pid/TheApp/RedPitaya_board/.

[10] Lorencon R. (1996). Elektronski Elementi in vezja; Narodna in Univerzitetna Knižnjica.

[11] Bizjak G., Kobav M.B., Prelovšek M., Tomažič S. (2014). Razsvetljava.

[12] Keitz H.A.E. (1971). Light Calculations and Measurements: An Introduction to the System of Quantities and Units in Light-Technology and to Photometry.

